# Reply to: Large influence of atmospheric vapor pressure deficit on ecosystem production efficiency

**DOI:** 10.1038/s41467-022-29010-3

**Published:** 2022-03-29

**Authors:** Laibao Liu, Lukas Gudmundsson, Mathias Hauser, Sonia I. Seneviratne

**Affiliations:** Institute for Atmospheric and Climate Science, ETH Zurich, Zurich, Switzerland

**Keywords:** Carbon cycle, Biogeochemistry

**replying to** Lu et al. *Nature Communications* 10.1038/s41467-022-29009-w (2022)

We read with interest the comments by Lu et al. regarding our study of disentangling soil moisture (SM) and vapor pressure deficit (VPD) impacts on ecosystem production (GPP) globally^1^. The additional analysis provided by Lu et al. contributes to the understanding of the effects of dryness stress on light use efficiency (LUE). However, Lu et al.’s commentary fails to recognize that: (i) LUE is only a contributing factor to GPP, and LUE alone cannot represent the overall GPP response to dryness; (ii) there is strong evidence for SM impacts on LUE as well; (iii) the core metric used by Lu et al. in their analysis is not available from primary observations globally. In the following, we provide additional background on each of these points.

## Light use efficiency alone cannot represent GPP’s response to dryness

Lu et al. employ a similar method as in our study (Liu et al.^1^, hereafter L20) to analyze the LUE relationship with water availability and they suggest based on their analyses that the estimate of the VPD impacts on GPP are underestimated in L20. Based on their comment, it appears that the authors have a misunderstanding regarding the scope of the L20 study since we investigate dryness stress on GPP, rather than on LUE. Following Monteith theory^2^, GPP is typically formulated as the product of incident photosynthetically active radiation (PAR), the fraction of absorbed PAR (fPAR), and LUE, such that

$${{{{{\rm{GPP}}}}}}={{{{{\rm{PAR}}}}}}\times {{{{{\rm{fPAR}}}}}}\times {{{{{\rm{LUE}}}}}}$$

In particular, LUE indicates the efficiency of translating absorbed photosynthetically active radiation (in energy units) to final tissue growth (in biomass units). Consequently, although LUE is an essential contributor to GPP (linked to plant physiological response or production efficiency), it cannot be equated with GPP. Hence, independently of whether LUE is more limited by SM or VPD, this relationship alone cannot lead to the conclusion that GPP has the same dependence on SM or VPD. For instance, in addition to LUE, fPAR is strongly modulated by dryness and can capture the ecosystem structure changes induced by dryness, such as leaf wilting or turgor loss. Consequently, fPAR alone contributes largely to the reduction in GPP induced by dryness, which is already well documented^3–6^. In addition, in the original paper^1^, we already tested the possible influence from PAR on the results by standardizing solar-induced fluorescence (SIF) by PAR (i.e., replacing SIF with SIF/PAR), which confirmed the robustness of our findings (see Supplementary Fig. 12 in L20). However, Lu et al. only rely on a constituent component of GPP (i.e., LUE) to assess impacts of dryness on vegetation, and their results do not allow to assess the full GPP response to dryness.

## Previously published evidence for SM impacts on LUE

Although the investigation of SM and VPD stress on LUE lies beyond the scope of L20, we note that existing literature that was not referred to by Lu et al. highlights the impacts of SM on LUE as well^7,8^. It has for example been shown that inter-annual variations of LUE are more strongly linked to water-balance components than to VPD^7^. Furthermore, recent investigations have identified substantial SM effects on LUE by utilizing observations from a large number of eddy covariance sites^8,9^. Contrasting these results with the analysis of Lu et al. highlights that whether LUE is more limited by SM or VPD at the global scale is still an open question.

## Caveats of data analyzed by Lu et al.

L20 analyzed SIF which is a direct indicator of GPP and is available as primary observation. In contrast, Lu et al. base their analyses on LUE, which is a derived quantity that has to be estimated using several data sets. In particular, Lu et al. approximate LUE using fluorescence quantum yield (SIF_yield_ = SIF/(PAR*fPAR)) which inherits observational uncertainty of all contributing data (i.e., SIF, PAR, and fPAR). For instance, fPAR is known to be affected by cloud and aerosol contaminations and is biased in high biomass regions^10^. We acknowledge the additional effort made by Lu et al. to validate this LUE estimate using the global FLUXCOM GPP data^11^. However, while FLUXCOM provides one of the most comprehensive global GPP estimates, it is also important to stress that design-choices underlying this machine-learning based product (e.g., related to the choice of explanatory variables) may introduce biases in subsequent analysis, which were not discussed by Lu et al. In particular, we note that this data product is known to underestimate the influence of water on global carbon fluxes^12^. Based on the foregoing, we conclude that the global estimates of LUE by Lu et al. are subject to uncertainties, weakening the conclusions that can be drawn from their analysis.

### Conclusion

The L20 global estimate of dryness stress on GPP is accurately derived and the Lu et al. analysis is not directly relevant for the evaluation of these results. We appreciate the authors’ interest in our study and the fact that they employ the L20 method to look into dryness effects on LUE. Further investigation of dryness stress on GPP’s mechanic components globally could be appealing and is anticipated to further advance the current understanding of terrestrial carbon–water coupling.

## Data Availability

No new data were generated for this response.

## Code availability

No new codes were generated for this response.
